# FNDC5 alleviates oxidative stress and cardiomyocyte apoptosis in doxorubicin-induced cardiotoxicity via activating AKT

**DOI:** 10.1038/s41418-019-0372-z

**Published:** 2019-06-17

**Authors:** Xin Zhang, Can Hu, Chun-Yan Kong, Peng Song, Hai-Ming Wu, Si-Chi Xu, Yu-Pei Yuan, Wei Deng, Zhen-Guo Ma, Qi-Zhu Tang

**Affiliations:** 1 Department of Cardiology, Renmin Hospital of Wuhan University, 430060 Wuhan, PR China; 2 Cardiovascular Research Institute of Wuhan University, 430060 Wuhan, PR China; 3 Hubei Key Laboratory of Cardiology, 430060 Wuhan, PR China

**Keywords:** Autocrine motility factor, Cardiomyopathies

## Abstract

Oxidative stress and cardiomyocyte apoptosis play critical roles in doxorubicin (DOX)-induced cardiotoxicity. Previous studies indicated that fibronectin type III domain-containing 5 (FNDC5) and its cleaved form, irisin, could preserve mitochondrial function and attenuate oxidative damage as well as cell apoptosis, however, its role in DOX-induced cardiotoxicity remains unknown. Our present study aimed to investigate the role and underlying mechanism of FNDC5 on oxidative stress and cardiomyocyte apoptosis in DOX-induced cardiotoxicity. Cardiomyocyte-specific FNDC5 overexpression was achieved using an adeno-associated virus system, and then the mice were exposed to a single intraperitoneal injection of DOX (15 mg/kg) to generate DOX-induced cardiotoxicity. Herein, we found that FNDC5 expression was downregulated in DOX-treated murine hearts and cardiomyocytes. *Fndc5* deficiency resulted in increased oxidative damage and apoptosis in H9C2 cells under basal conditions, imitating the phenotype of DOX-induced cardiomyopathy in vitro, conversely, FNDC5 overexpression or irisin treatment alleviated DOX-induced oxidative stress and cardiomyocyte apoptosis in vivo and in vitro. Mechanistically, we identified that FNDC5/Irisin activated AKT/mTOR signaling and decreased DOX-induced cardiomyocyte apoptosis, and moreover, we provided direct evidence that the anti-oxidant effect of FNDC5/Irisin was mediated by the AKT/GSK3β/FYN/Nrf2 axis in an mTOR-independent manner. And we also demonstrated that heat shock protein 20 was responsible for the activation of AKT caused by FNDC5/Irisin. In line with the data in acute model, we also found that FNDC5/Irisin exerted beneficial effects in chronic model of DOX-induced cardiotoxicity (5 mg/kg, i.p., once a week for three times, the total cumulative dose is 15 mg/kg) in mice. Based on these findings, we supposed that FNDC5/Irisin was a potential therapeutic agent against DOX-induced cardiotoxicity.

## Introduction

Despite its effectiveness in the treatment of various human cancers, the chemotherapeutic application of doxorubicin (DOX) is largely limited for the cause of life-threatening cardiotoxicity [1, 2]. Multiple factors have been substantiated to be involved in the pathogenesis of DOX-induced cardiotoxicity, including DNA/RNA/protein synthesis inhibition, autophagy dysregulation and the disturbance of intracellular calcium homeostasis, however, emerging studies proposed indispensable roles of reactive oxygen species (ROS) overproduction and cardiomyocyte apoptosis in DOX-induced myocardial damage [3–5]. Excessive ROS induces oxidative damage to biological macromolecules, including lipids, proteins and DNA, and disrupts cellular membrane integrity and function [6]. Moreover, DOX-evoked oxidative stress can directly elicit massive cardiomyocyte apoptosis via both extrinsic and intrinsic apoptotic pathways and cause severe cardiac dysfunction [7, 8]. Previous studies also demonstrated that DOX could induce apoptosis via mechanisms that do not directly involve ROS production and oxidative stress [8]. Our recent study also revealed that suppression of cardiomyocyte apoptosis significantly attenuated DOX-induced cardiac dysfunction [9]. Therefore, an intensive understanding of the pathogenesis of DOX-induced oxidative stress and cardiomyocyte apoptosis, and the identification of novel therapeutic targets are urgently needed.

Protein kinase B (PKB/AKT) is a serine/threonine protein kinase involved in the regulation of cell survival, proliferation, and metabolism and is inhibited in DOX-treated murine hearts [10]. Increasing studies proved that activation of AKT prevented cardiomyocyte apoptosis in response to DOX, whereas AKT inhibition exaggerated DOX-induced cardiomyocyte apoptosis and cardiac dysfunction [11, 12]. The mammalian target of rapamycin (mTOR) is a classical and important downstream mediator in AKT signaling pathway, which exerts beneficial role in regulating protein synthesis and cell survival via P70 S6 kinase (P70) and 4E-binding protein 1 (4EBP1) [13]. Zhu et al. found that mTOR inhibition was the major contributor to DOX-triggered myocardial mass loss and cardiac dysfunction, and overexpression of constitutively active mTOR could protect DOX-induced cardiotoxicity [14]. In addition to the role in regulating cell survival, current available data suggested that AKT also played a critical role in relieving oxidative stress via deactivating glycogen synthase kinase 3β (GSK3β), which thereby decreases FYN nuclear translocation-mediated NF-E2-related factor 2 (Nrf2) nuclear export and degradation [15–17]. Considering the beneficial role in DOX-induced cardiotoxicity, it is important to unearth a novel positive regulator of AKT.

Fibronectin type III domain-containing protein 5 (FNDC5, also known as FRCP2 and PeP) is a glycosylated transmembrane protein, with a signal peptide, two fibronectin domains and one hydrophobic domain inserted into the cell membrane, that can be cleaved and released as irisin [18]. FNDC5/Irisin was traditionally found in muscle and regarded as a myokine that drives brown-fat-like development of white fat and thermogenesis, which was suggested as a therapeutic agent for human metabolic diseases [19]. Liu et al. found that *Fndc5* deficiency aggravated whereas FNDC5 overexpression prevented obesity-related hyperlipemia, hepatic lipid accumulation, and impaired fatty acid oxidation and autophagy in the liver [20]. Except for the beneficial role in metabolic disorders, recent studies also implicated that FNDC5/Irisin was involved in regulating various cardiovascular diseases, such as atherosclerosis, hypertension, myocardial ischemia/reperfusion injury, and cardiac hypertrophy [21–24]. Besides, numerous researches verified that FNDC5 overexpression or irisin supplementation could preserve mitochondrial function and attenuate oxidative damage as well as cell apoptosis [25, 26]. Based on these findings, we hypothesized that FNDC5/Irisin may be a promising candidate for the treatment of DOX-induced cardiotoxicity.

## Methods and materials

### Antibodies and reagents

Antibodies against the following proteins were purchased from Cell Signaling Technology (Danvers, MA, USA): BAX (1:1000), cleaved-Caspase3 (C-Caspase, 1:1000), total Caspase3 (T-Caspase3, 1:1000), total AKT (T-AKT, 1:1000), phosphorylated AKT (P-AKT, 1:1000), T-mTOR (1:1000), P-mTOR (1:1000), T-P70 (1:1000), P-P70 (1:1000), T-ribosomal protein S6 (T-S6, 1:1000), P-S6 (1:1000), T-4EBP1 (1:1000), P-4EBP1 (1:1000), T-glycogen synthase kinase 3β (T-GSK3β, 1:1000), P-GSK3β (1:1000), 4-Hydroxynonenal (4-HNE, 1:200 for staining), and glyceraldehyde 3-phosphate dehydrogenase (GAPDH, 1:1000). Antibodies for FNDC5 (1:1000 for western blot, 1:100 for staining), p67phox (1:1000), superoxide dismutase 1 (SOD1, 1:1000), SOD2 (1:1000), B-cell lymphoma 2 (BCL-2, 1:1000), Nrf2 (1:1000), heme oxygenase-1 (HO-1, 1:1000), Kelch-like ECH-associated protein 1 (Keap1, 1:1000), and heat shock protein 20 (HSP20, 1:1000) were purchased from Abcam (Cambridge, UK). Anti-T-FYN (1:200), anti-P-FYN (1:200), and anti-T-proliferating cell nuclear antigen (PCNA, 1:200) were obtained from Santa Cruz Biotechnology (Dallas, TX, USA). The secondary antibody used for western blot was purchased from LI-COR Biosciences, whereas anti-rabbit/mouse EnVision^TM+^/HRP reagent used for immunohistochemistry was obtained from Gene Technology (Shanghai, China). DOX, irisin, AKT inhibitor (AKT i), rapamycin (Rapa) and dexrazoxane (DEX) were purchased from Sigma-Aldrich (St. Louis, MO, USA). Dihydroethidium (DHE) was obtained from Keygen Biotech, and 2′,7′-dichlorodihydrofluorescein diacetate (DCFH-DA), malondialdehyde (MDA) assay kit, glutathione (GSH) assay kit, total SOD assay kit and NADPH oxidase assay kit were all purchased from Nanjing Jiancheng Bioengineering Institute (Nanjing, China). Phosphoinositide 3-kinase (PI3K) activity ELISA assay kit was obtained from Echelon Biosciences Inc. ApopTag® Plus In Situ Apoptosis Fluorescein Detection Kit was purchased from Millipore (Billerica, MA, USA) and the cell counting kit-8 (CCK-8) was obtained from Dōjindo Laboratories (Kumamoto, Japan).

### Animals and treatments

All animal care and experimental procedures were in compliance with the Guidelines for the Care and Use of Laboratory Animals published by the United States National Institutes of Health (NIH Publication, revised 2011) and approved by the Animal Care and Use Committee of Renmin Hospital of Wuhan University. C57BL/6 male mice (8–10 weeks old, 23.5–27.5 g) were purchased from the Institute of Laboratory Animal Science, Chinese Academy of Medical Sciences (Beijing, China) and were subjected to an adaptive feeding for 1 week before the study commenced. All mice were maintained under specific pathogen-free, environmentally controlled (Temperature: 20–25 °C; Humidity: 50 ± 5%) barrier conditions in individual ventilated cages and were fed with sterile food and water ad libitum. To specifically overexpress FNDC5 in the myocardium, mice received a single intravenous injection of adeno-associated virus 9 (AAV9) carrying human FNDC5 under the cTnT promoter (AAV9-FNDC5) or a negative control (AAV9-NC) via the tail vein at a concentration of 1 × 10^11^ viral genome per mouse [9]. The AAV9-FNDC5 and AAV9-NC were generated by Hanbio Biotechnology Co. (Shanghai, China). Four weeks post-AAV9 injection, the mice were exposed to a single intraperitoneal injection of DOX (15 mg/kg) to generate DOX-induced cardiotoxicity or equal volume of normal saline (NS) as a control referring to our previous study [9]. These mice were observed daily and weighed every 2 days, and then were sacrificed with an overdose of sodium pentobarbital (200 mg/kg; i.p.) after 8-day DOX insult. Murine hearts together with the tibia were collected to calculate the heart weight/tibia length ratios (HW/TL). To verify the hypothesis that the beneficial roles of FNDC5 were dependent on the activation of AKT, mice were intraperitoneally injected with AKT i (20 mg/kg/day) for consecutive 14 days from the 6th day before DOX insult [27]. For mTOR activity inhibition, mice were daily treated with rapamycin (5 mg/kg/day) for 14 days as AKT i via intraperitoneal injection according to previous studies [20, 28]. To verify the role of Nrf2 and HSP20 in vivo, mice were exposed to a single intravenous injection of AAV9 carrying small hairpin RNA against Nrf2 (sh *Nrf2*) or HSP20 (sh *Hsp20*) or their corresponding negative control (sh *RNA*) 2 weeks before FNDC5 overexpression. To investigate the therapeutic effect of irisin, mice were subcutaneously infused with either saline or irisin (12 nmol/kg/day) for 14 days from the 6th day before DOX injection via osmotic minipumps (Alzet model 2004, Alza Corp) as previously described [29, 30]. In addition, mice were intraperitoneally injected with a weekly dose of 60 mg/kg DEX for two times, with the first injection at the 6th day before DOX injection, and the second one at the second day after DOX treatment [31].

To enhance the clinical impact of our current work, mice were injected intraperitoneally with DOX (5 mg/kg, once a week, the total cumulative dose is 15 mg/kg) for three times to generate chronic model of DOX-induced cardiotoxicity [32]. After 6 weeks from the first injection of DOX, cardiac function was evaluated and murine hearts were collected for further detection. Four weeks before DOX injection, mice received a single intravenous injection of AAV9-FNDC5 to overexpress FNDC5 in myocardium or AAV9-NC as a control. All these mice were observed daily and the survival rate was calculated every week. To compare the effect of irisin and DEX in chronic model, mice with irisin protection were pretreated with irisin (12 nmol/kg/day) for 6 days and lasting for another 6 weeks, whereas mice with DEX administration were intraperitoneally injected with a weekly dose of 60 mg/kg DEX for seven times [29–31]. Considering the fact that long-term use of high-doses of DEX resulted in severe side effects, we then investigated whether combined use of DEX with irisin (6 nmol/kg/day) could decrease the usage of DEX. In this experiment, mice were exposed to DEX (60 mg/kg/week), DEX (30 mg/kg/week), or DEX + irisin (30 mg/kg/week for DEX, 6 nmol/kg/day for irisin) in the presence of chronic DOX insult.

### Echocardiography and hemodynamics

Transthoracic echocardiography was performed according to our previous studies, and echocardiographic parameters were averaged from three to five cardiac cycles [33–37]. Particular attention was given not to bring excessive pressure to the chest, which could cause bradycardia and deformation of the heart. Invasive hemodynamic monitoring was performed by PowerLab system (AD Instruments Ltd., Oxford, UK) using a 1.4-French Millar pressure-volume catheter (SPR-839; Millar Instruments, Houston, TX).

### Western blot and quantitative real-time PCR

Western blot and quantitative real-time PCR were performed referring to our previous articles [33–37]. Nuclear protein fractions were separated by a commercial kit as our previously described and were normalized to PCNA [9, 33]. Total RNA was extracted using TRIzol reagent and reverse transcribed with Maxima First Strand cDNA Synthesis Kit [Roche (Basel, Switzerland), 04896866001]. The expression level of each individual transcript was normalized to *Gapdh*.

### Immunohistochemistry and TdT-mediated dUTP nick end-labeling (TUNEL) staining

Immunohistochemistry staining was performed according to our previous studies [38, 39]. Endogenous peroxidase and the nonspecific binding of the antibody were blocked with 3% hydrogen peroxide or 10% goat serum, respectively. Sections were determined by the light microscopy (Nikon (Tokyo, Japan), H550L) in a blinded manner. TUNEL staining was performed to detect cell apoptosis both in vivo and in vitro according to the manufacturer’s instructions using a commercially available kit, and the images were captured by a special OLYMPUS DX51 fluorescence microscope (Tokyo, Japan).

### Cell culture and treatments

H9C2 cells were purchased from the Cell Bank of the Chinese Academy of Sciences (Shanghai, China) and were cultured in Dulbecco’s modified Eagle’s medium with 10% fetal bovine serum. After synchronization for 24 h, cells were treated with irisin (20 nmol/L) in the presence or absence of DOX (1 μmol/L) for 24 h [9, 24]. For AKT or mTOR inhibition, H9C2 cells were pretreated with AKT i (1 μmol/L), or rapamycin (50 nmol/L) for 30 min [20, 24, 38]. To knockdown the expression of FNDC5 or Nrf2, cells were transfected with scramble small interfering RNA (si *RNA*, 50 nmol/L), si *Fndc5* (50 nmol/L) or si *Nrf2* (50 nmol/L) for 4 h using Lipo6000^TM^ transfection reagent according to the manufacturer’s protocol (RiboBio Co. Ltd, Guangzhou, Guangdong, China), and then were cultured in normal medium for 24 h before the next treatment [9, 33].

### Oxidative stress detection and cell viability

ROS production was evaluated by DHE staining in vivo and DCFH-DA staining in vitro [38, 40]. Briefly, cryosections of fresh heart samples or coverslips were stained with DHE (5 μmol/L) or DCFH-DA (5 μmol/L) in the dark at 37 °C for 30 min, and then were visualized in a blinded manner under an Olympus IX53fluorescence microscope. To further assess oxidative stress level, we measured the content of MDA, GSH, total SOD activity and NADPH oxidase activity in the myocardium or H9C2 cells according to our previous study by the commercially available kits [38]. Cell viability was determined using the CCK-8 assay kit according to the manufacturer’s protocol as described previously [9, 38].

### Biochemical determination

Serum was collected and the concentrations of cardiac isoform of Tropnin T (cTnT), lactate dehydrogenase (LDH) and creatine kinase isoenzymes (CK-MB) were measured by an automatic biochemical analyzer (ADVIA® 2400, Siemens Ltd., China) [41].

### Statistical analysis

All data in this research were expressed as mean ± standard error of the mean (SEM) and analyzed by SPSS22.0 software. One way analysis of variance (ANOVA) followed by Tukey post hoc test was performed when comparing multiple groups, whereas differences in two groups were evaluated using unpaired Student’s *t*-test. Survival data were assessed by the Kaplan–Meier method and survival curves were compared using the Mantel–Cox log-rank test. A *P* value <0.05 was considered statistically significant.

## Results

### FNDC5 attenuated DOX-induced cardiac dysfunction in mice

Previous studies reported that FNDC5 was mainly expressed in skeletal muscle [18], however, we found that the relative mRNA expression of *Fndc5* was more abundant in the myocardium than that in skeletal muscle, which was downregulated in response to DOX administration (Fig. S1a–b). Immunohistochemistry staining showed that FNDC5 in the heart was mainly localized to cardiomyocytes, and the protein level was also significantly decreased in DOX-treated hearts, which was further confirmed by the western blot results (Fig. S1c–d). In addition, DOX incubation suppressed FNDC5 expression in isolated cardiomyocytes (Fig. S1e).

**Fig. 1:**
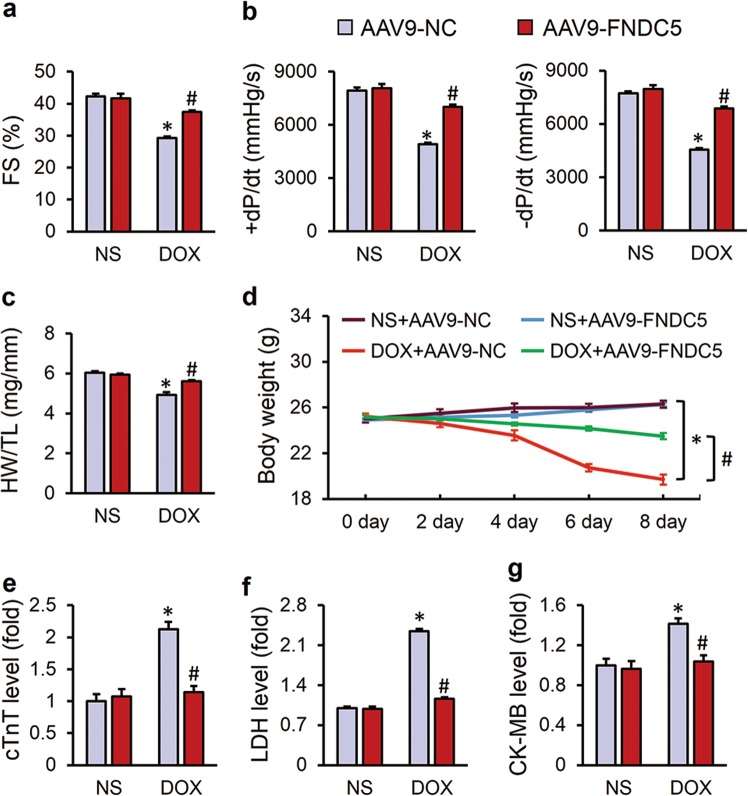
FNDC5 attenuated doxorubicin (DOX)-induced cardiac dysfunction in mice. **a** Fractional shortening (FS) of mice as determined via echocardiography 8 days after DOX injection (*n* = 8). **b** Hemodynamic parameter of mice with or without FNDC5 overexpression (*n* = 8). **c** Statistical results of the heart weight/tibia length ((HW/TL) (*n* = 8). (**d**) Body weight alterations (*n* = 8). **e**–**g** Biochemical determination of cTnT, LDH, CK-MB serum levels (*n* = 10). Values represent the mean ± SEM. *P < 0.05 versus the corresponding normal saline (NS) group mice injected with negative control (NC) adeno-associated virus 9, ^#^*P* < 0.05 versus DOX-treated mice injected with AAV9-NC

To explore the role of FNDC5 in DOX-induced cardiotoxicity, we specifically overexpressed FNDC5 in myocardium via a single injection of an AAV9 carrying FNDC5 under the cTnT promoter. As shown in Fig. S1f, the myocardial mRNA level of FNDC5 was markedly increased in mice infected with AAV9-FNDC5. Besides, myocardial FNDC5 protein level was restored to normal level in the presence of DOX, which is moderate and efficacious, therefore we selected this time for the next experiments (Fig. S1g). As shown in Fig. 1a, b, FNDC5 overexpression prevented DOX-induced cardiac dysfunction, as indicated by the preserved fractional shortening (FS) and ±d*p*/d*t*. Besides, we also observed that DOX injection decreased the ratio of heart weight to tibia length (HW/TL), which were significantly attenuated by FNDC5 overexpression (Fig. 1c). Body weight loss due to chemotherapeutic agents and cachexia syndrome predicts bad prognosis in cancer patients via compromising functions of skeletal muscle, adipose tissue and also internal organs, including the liver, kidneys, lungs, especially the heart [42]. Previous studies indicated that DOX application significantly decreased the body weight in cancer patients [43], but intriguingly, we found that cardiac-specific overexpression of FNDC5 attenuated DOX-induced body weight loss in mice, which raises the possibility for its clinical use (Fig. 1d). Myocardial injury was also assessed by the serum levels of cTnT, LDH, and CK-MB, and we observed that FNDC5 could alleviate DOX-induced myocardial injury (Fig. 1e–g). Collectively, we concluded that FNDC5 overexpression attenuated DOX-induced cardiotoxicity in mice.

### FNDC5 protected heart from oxidative damage in response to DOX insult

**Fig. 2:**
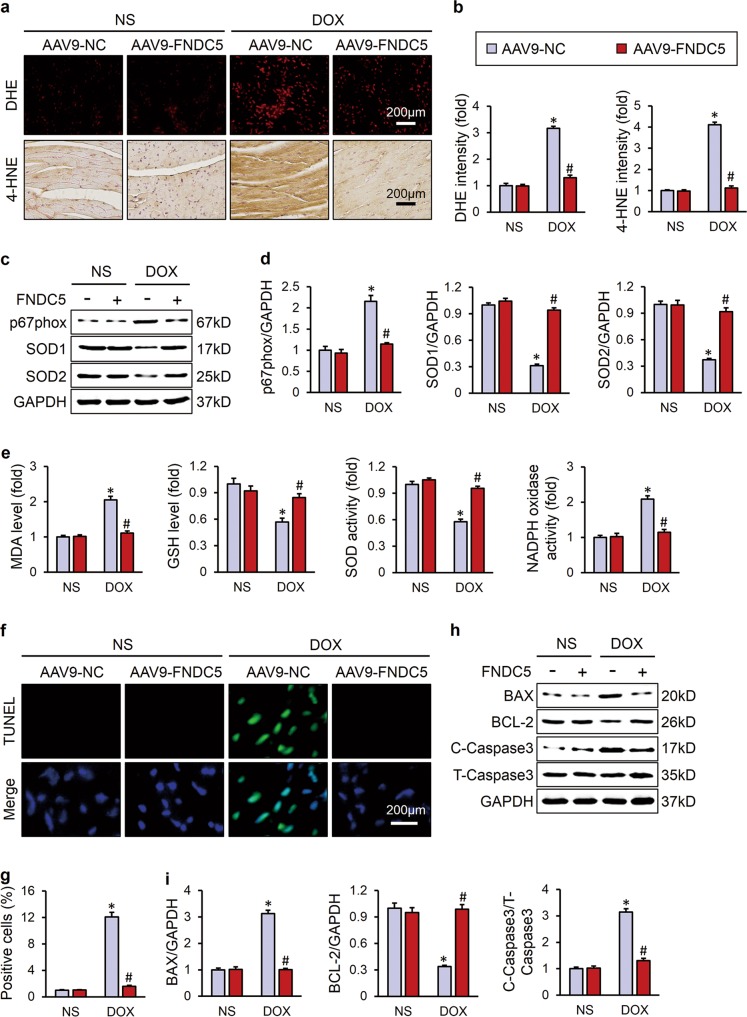
FNDC5 suppressed oxidative stress and cardiomyocyte apoptosis in DOX-treated mice. **a**, **b** Representative DHE, 4-HNE staining images and the quantitative results (*n* = 6). **c**, **d** Western blots and statistical results (*n* = 6). **e** Quantitative results of myocardial MDA, GSH levels and SOD, NADPH oxidase activities (*n* = 6). **f**, **g** TUNEL staining and the quantitative results (*n* = 8). **h**, **i** Western blots and the statistical results of apoptosis-related proteins (*n* = 6). Values represent the mean ± SEM. *P < 0.05 versus the corresponding normal saline (NS) group mice injected with negative control (NC) adeno-associated virus 9, ^#^*P* < 0.05 versus DOX-treated mice injected with AAV9-NC

Oxidative damage is suggested to be the main cause of DOX-induced cardiotoxicity, thus we detected oxidative stress level in the heart via DHE and 4-HNE staining. DOX injection resulted in increased oxidative stress in the heart, and FNDC5 overexpression almost completely inhibited ROS production (Fig. 2a, b). In addition, western blot showed that FNDC5 overexpression significantly decreased the NADPH oxidase subunit p67phox and increased SOD1 and SOD2 expression in DOX-treated hearts (Fig. 2c, d). Consistent with the molecular alterations, we found that FNDC5 overexpression reduced the abnormal MDA level and NADPH oxidase activity, and preserved GSH level and total SOD activity (Fig. 2e).

### FNDC5 suppressed cardiomyocyte apoptosis in DOX-treated mice

Excessive ROS production and DOX itself could both induce cardiomyocyte apoptosis and contributed to the progression of cardiac dysfunction, and we then assessed the role of FNDC5 in cardiomyocyte apoptosis. As shown in Fig. 2f, g, DOX injection resulted in distinct cardiomyocyte apoptosis in vivo, and overexpression of FNDC5 mitigated this pathological alteration. The inhibitory effects of FNDC5 on cardiomyocyte apoptosis were further confirmed by western blot results showing that FNDC5 decreased the expression of BAX, C-Caspase3 and increased the BCL-2 level (Fig. 2h, i).

### FNDC5 alleviated DOX-induced oxidative stress and cardiomyocyte apoptosis in vitro

**Fig. 3:**
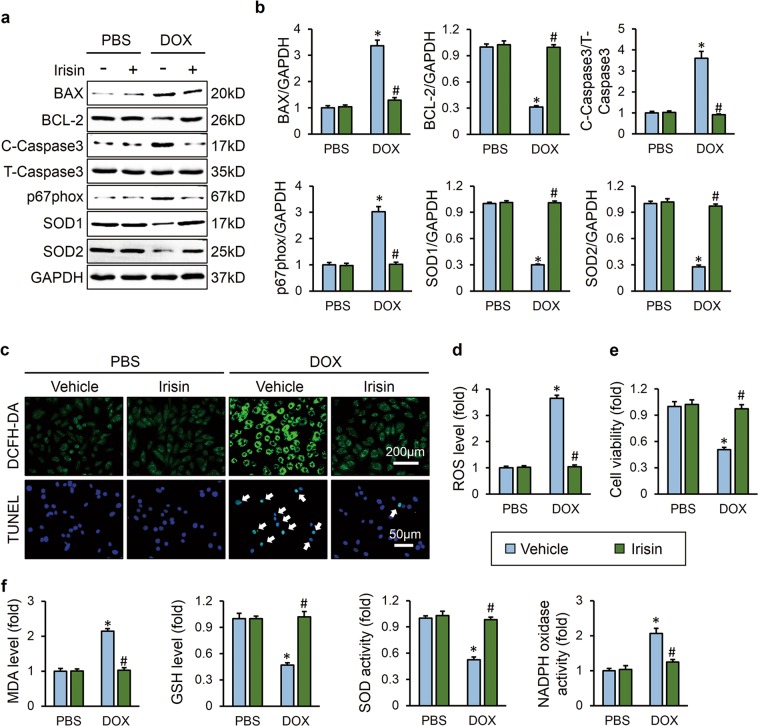
FNDC5 alleviated DOX-induced oxidative stress and cardiomyocyte apoptosis in vitro. **a**, **b** Western blots and statistical results (*n* = 6). **c**, **d** DCFH-DA staining, TUNEL staining and the quantitative results of ROS level (*n* = 6). **e** Cell viability detected by CCK-8 assay (*n* = 6). **f** Quantitative results of MDA, GSH levels and SOD, NADPH oxidase activities in cultured H9C2 cells (*n* = 6). Values represent the mean ± SEM. *P < 0.05 versus PBS group treated with control vehicle, ^#^*P* < 0.05 versus DOX-treated H9C2 cells with irisin protection

To further verify the beneficial role of FNDC5 on DOX-induced oxidative stress and cardiomyocyte apoptosis, we treated H9C2 cells with irisin, a cleaved and secreted fragment of FNDC5, in the presence or absence of DOX in vitro. In line with the data in vivo, we found that irisin showed no effect at baseline, but significantly attenuated DOX-induced upregulation of BAX, C-Caspase3, p67phox and the downregulation of BCL-2, SOD1, and SOD2, and exerted protective effects on oxidative stress and apoptosis in vitro (Fig. 3a, b). DCFH-DA staining and biochemical analysis further confirmed that irisin treatment alleviated DOX-induced oxidative damage to cardiomyocytes (Fig. 3c–f). TUNEL staining as well as CCK-8 assay results indicated that DOX incubation caused massive apoptosis in H9C2 cells, which was notably reversed by irisin protection (Fig. 3c, e). Next, we investigated whether *Fndc5* deficiency affected oxidative stress and cardiomyocyte apoptosis in vitro. We knocked down the expression of FNDC5 in H9C2 cells and the efficiency was confirmed by western blot (Fig. S2a, f). Downregulation of FNDC5 resulted in increased cardiomyocyte apoptosis at baseline, as evidenced by the decrease of BCL-2 protein level, cell viability and increase of BAX, C-Caspase3 level, TUNEL positive cells (Fig. S2a–c, e). Data from western blot and DCFH-DA staining also suggested that *Fndc5* deficiency increased oxidative stress level and ROS production in basal conditions (Fig. S2a–d). All these results implied that FNDC5 alleviated DOX-induced oxidative stress and cardiomyocyte apoptosis in vitro.

### AKT/mTOR signaling was responsible for FNDC5-mediated protective role on cardiomyocyte apoptosis

**Fig. 4:**
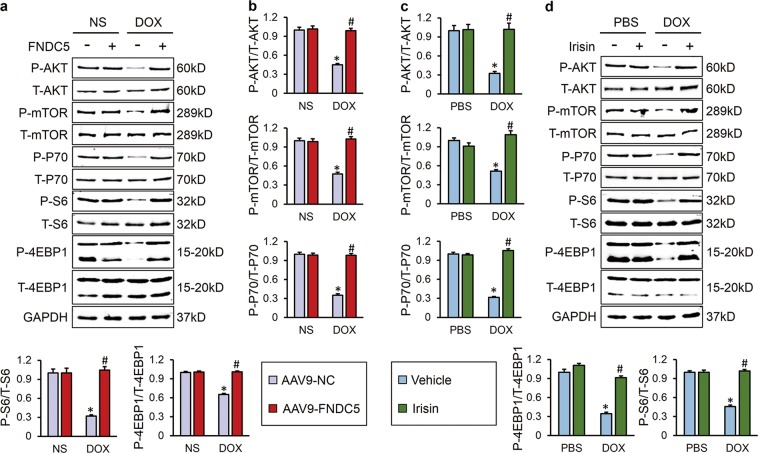
FNDC5 activated AKT/mTOR signaling pathway in vivo and in vitro. **a**, **b** Western blots and quantitative results in murine hearts (*n* = 6). **c**, **d** Western blots and statistical results in cultured H9C2 cells (*n* = 6). Values represent the mean ± SEM. In figure (**a**, **b**), *P < 0.05 versus the corresponding normal saline (NS) group mice injected with negative control (NC) adeno-associated virus 9, ^#^*P* < 0.05 versus DOX-treated mice injected with AAV9-NC. In figure (**c**, **d**), *P < 0.05 versus PBS group treated with control vehicle, ^#^*P* < 0.05 versus DOX-treated H9C2 cells with irisin protection

**Fig. 5:**
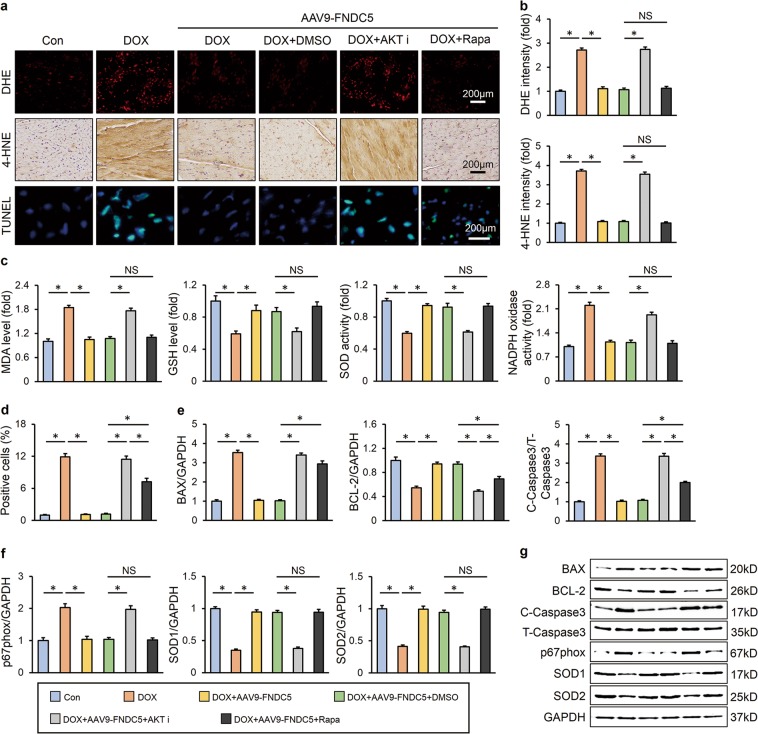
AKT/mTOR signaling was responsible for FNDC5-mediated protective role on cardiomyocyte apoptosis. **a** Representative DHE, 4-HNE, TUNEL staining images (*n* = 6). **b** Quantitative results of DHE and 4-HNE intensity (*n* = 6). **c** Quantitative results of myocardial MDA, GSH levels and SOD, NADPH oxidase activities (*n* = 6). **d** Quantitative results of TUNEL-positive cells (*n* = 6). **e**–**g** Western blots and statistical results (*n* = 6). Values represent the mean ± SEM. *P < 0.05 versus the matched group. NS means no significance

AKT/mTOR is an important signal transduction pathway responsible for cell survival and apoptosis [10]. Previous studies proved that AKT/mTOR signaling was inhibited in DOX-treated hearts, and restoration of myocardial AKT blunted DOX-induced cardiac dysfunction [11]. Here, we observed that both myocardial overexpression of FNDC5 in vivo and irisin treatment in vitro mitigated DOX-induced inactivation of AKT/mTOR pathway, further confirmed by the increased phosphorylation of P70/S6 and 4EBP1 (Fig. 4a–d). To gain evidence that the protective effects of FNDC5 were mediated by AKT/mTOR activation, we treated mice with either AKT inhibitor or rapamycin. As shown in Fig. 5a–g, AKT inhibition almost completely abolished the beneficial role of FNDC5 on oxidative stress and cardiomyocyte apoptosis, whereas mTOR blockade by rapamycin only partly reversed the improvement of cardiomyocyte apoptosis by FNDC5 overexpression, with almost no effect on FNDC5-mediated anti-oxidant effects. In line with the data in vivo, we found that irisin lost both of the anti-oxidant and anti-apoptotic effects in cells treated with AKT inhibitor, whereas its anti-oxidant capacity was preserved in rapamycin-treated cells (Fig. S3a-f). Regarding the fact that mTOR is merely one of the downstream effector of AKT and mainly mediated the survival signals [14], we supposed that FNDC5 might exerted its anti-oxidant effect via a mechanism that is AKT-dependent, but mTOR-independent. Collectively, these data showed that AKT/mTOR signaling was responsible for FNDC5-mediated protective role on cardiomyocyte apoptosis.

### AKT/GSK3β/FYN/Nrf2 signaling was responsible for FNDC5-mediated protective role on oxidative damage

**Fig. 6:**
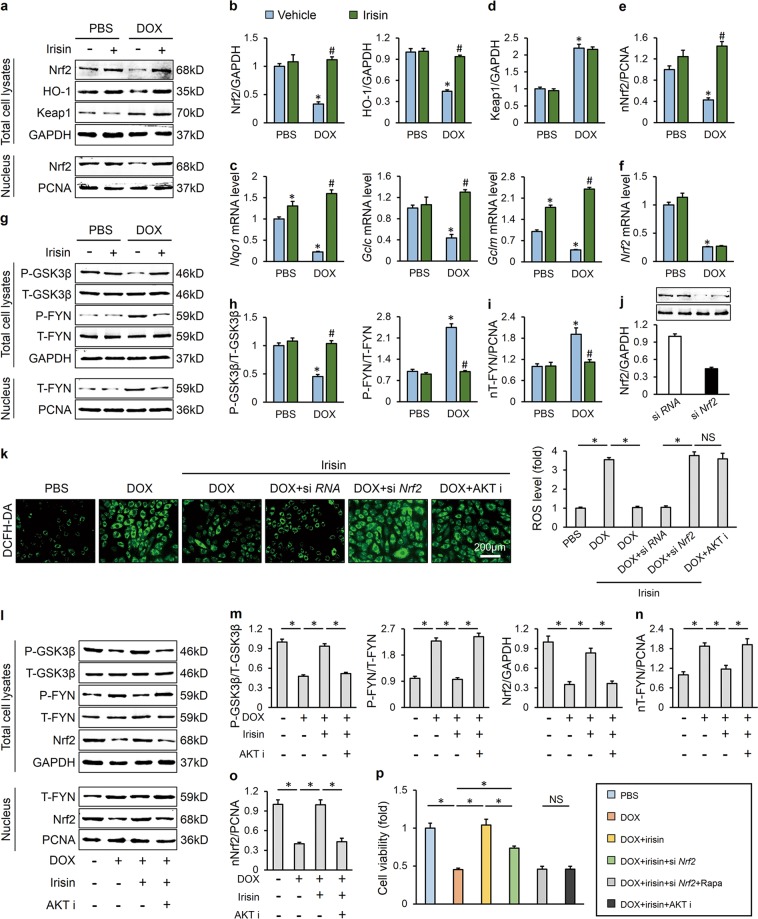
AKT/GSK3β/FYN/Nrf2 signaling was responsible for FNDC5-mediated protective role on oxidative damage. **a**, **b** Western blots and statistical results (*n* = 6). **c** Relative mRNA levels of *Nqo1*, *Gclc*, and *Gclm* in cultured H9C2 cells (*n* = 6). **d**, **e** Keap1 protein levels in total cell lysates and Nrf2 protein levels in nuclear lysates (*n* = 6). **f** Relative *Nrf2* mRNA level (*n* = 6). **g**, **h** Representative western blots (*n* = 6). **i** FYN protein levels in nuclear lysates (*n* = 6). **j** Efficiency of si *Nrf2* determined by western blots (*n* = 6). **k** DCFH-DA staining and the statistical data (*n* = 6). **l**–**o** Western blots and quantitative results (*n* = 6). **p** Cell viability detected by CCK-8 assay (*n* = 6). Values represent the mean ± SEM. *P < 0.05 versus the matched group. NS means no significance

Nrf2, a redox sensitive transcription factor, plays a pivotal role in redox homeostasis during oxidative stress [44]. Previous studies indicated that *Nrf2* deficiency exacerbated DOX-induced cardiotoxicity, whereas Nrf2 activation provided protection against cardiac dysfunction in response to DOX [7, 45]. Here, we found that irisin treatment blocked DOX-induced Nrf2 downregulation and preserved its transcriptional activity, as confirmed by the increased HO-1 protein level and *Nqo1*, *Gclc*, *Gclm* mRNA level (Fig. 6a–c). Keap1 is known to facilitate the ubiquitination and degradation of Nrf2 and regard as a major negative regulator of the antioxidant response [46]. However, we found that Keap1 expression was unaffected by irisin treatment (Fig. 6d). Consistent with the preserved transcriptional activity, we found that the protein level of Nrf2 in nucleus was increased with irisin incubation (Fig. 6a, e). As the mRNA level of Nrf2 was also unaltered, we speculated that irisin might inhibit the nuclear export and degradation of Nrf2 (Fig. 6f). Numerous studies have defined a role of AKT/GSK3β/FYN axis in the regulation of Nrf2 nuclear export [15–17]. And in view of the role of AKT in FNDC5-mediated anti-oxidant effect, we thus detected this axis in H9C2 cells. As shown in Fig. 6g–i, irisin treatment inactivated GSK3β and reduced FYN phosphorylation and its nuclear accumulation, which in turn decreased Nrf2 nuclear export and cytoplasmic degradation. In addition, *Nrf2* deficiency also blocked the beneficial effect of irisin on oxidative stress, which is similar as AKT inhibitor (Fig. 6j, k). Consistent with the data in vitro, we also observed that FNDC5 lost its anti-oxidant effect in *Nrf2*-deficient mice (Figure S4). To further verify the role of AKT in the regulation of Nrf2, we treated H9C2 cells with an AKT inhibitor, and found that AKT inhibition resulted in GSK3β activation and promoted FYN nuclear translocation-mediated Nrf2 export and degradation, and abrogated the beneficial effect of irisin (Fig. 6k–o). Intriguingly, we found that *Nrf2* deficiency partly abrogated irisin-mediated anti-apoptotic effect, nevertheless, *Nrf2* deficiency together with mTOR inhibition exhibited a similar outcome on cardiomyocyte apoptosis as AKT inhibition (Fig. 6p). These data confirmed that AKT/GSK3β/FYN/Nrf2 signaling was responsible for FNDC5-mediated protective role on oxidative damage.

### *HSP20* was involved in the activation of AKT caused by FNDC5

**Fig. 7:**
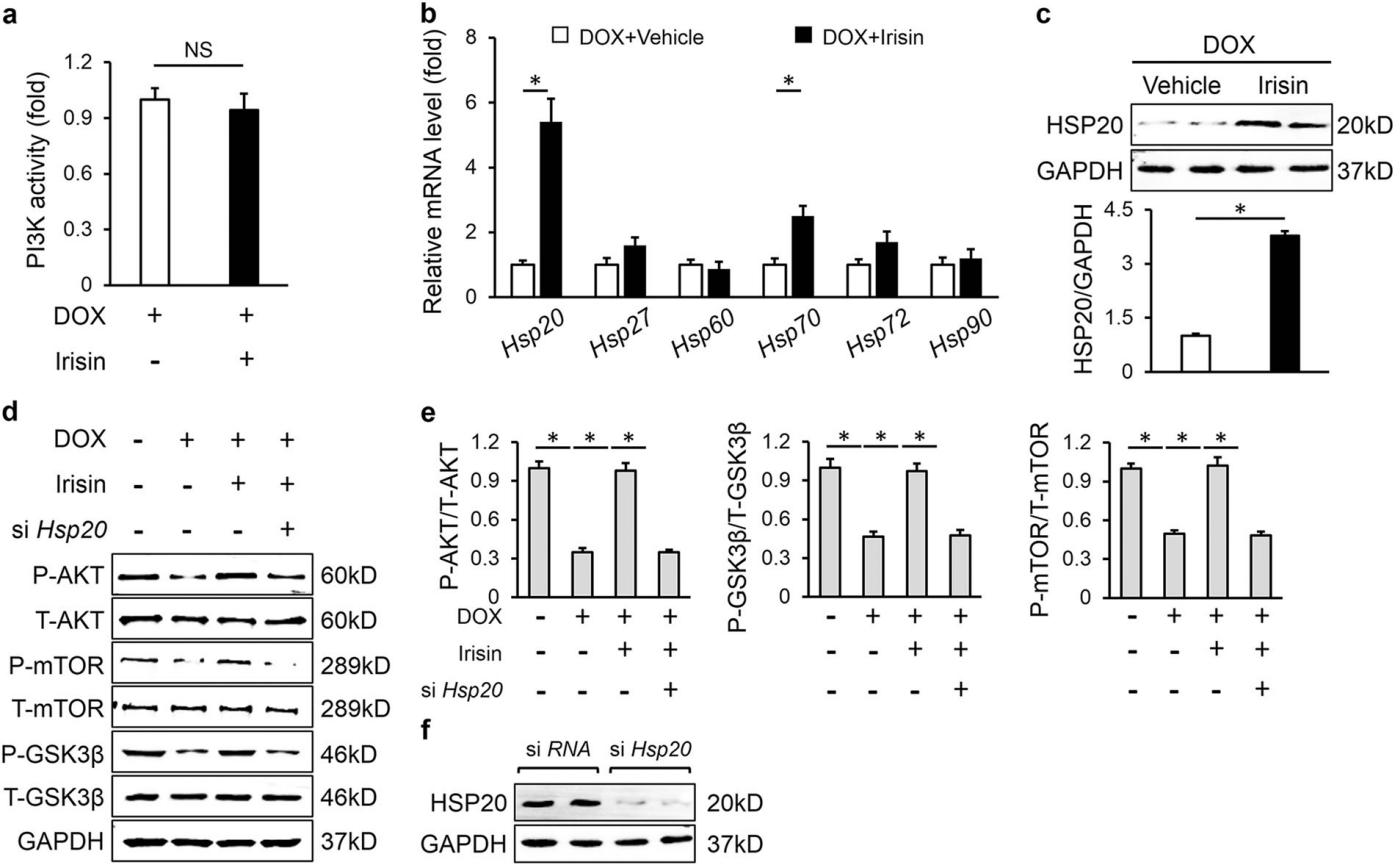
HSP20 was involved in the activation of AKT caused by FNDC5. **a** Quantitative results of PI3K activity (*n* = 5). **b** Relative mRNA levels (*n* = 6). **c** HSP20 protein levels and the statistical results (*n* = 6). **d**, **e** Western blots and quantitative data (*n* = 6). **f** Efficiency of si *Hsp20* detected by western blots (*n* = 6). Values represent the mean ± SEM. *P < 0.05 versus the matched group. NS means no significance

Next, we investigated the possible mechanism by which FNDC5 activated AKT. We first detected PI3K activity, because it is suggested as an upstream activator of AKT and previous studies reported that FNDC5 activated AKT via PI3K [29, 47]. Unexpectedly, we found that irisin unaltered PI3K activity in the presence of DOX in H9C2 cells (Fig. 7a). HSPs play important roles in cellular stress resistance and exert protective effects on DOX-induced cardiac dysfunction [48]. Real-time PCR results implied that irisin significantly increased the mRNA level of *Hsp20*, with a slight increase of *Hsp70* (Fig. 7b). Fan et al. previously demonstrated that HSP20 could interact with the phosphorylated AKT and prevent it from dephosphorylating by the phosphatase [12]. Herein, we observed that irisin also increased the protein level of HSP20 in response to DOX, whereas *Hsp20* silence reduced irisin-mediated activation of AKT, as confirmed by the two downstream effector, GSK3β and mTOR (Fig. 7c–f). Moreover, we also found that FNDC5 overexpression-induced activation of AKT was abolished in *Hsp20*-deficient mice (Fig. S5a-b). Thus, we concluded that FNDC5 activated AKT via upregulation of HSP20.

### FNDC5/Irisin was a potential therapeutic agent against DOX-induced cardiotoxicity

**Fig. 8:**
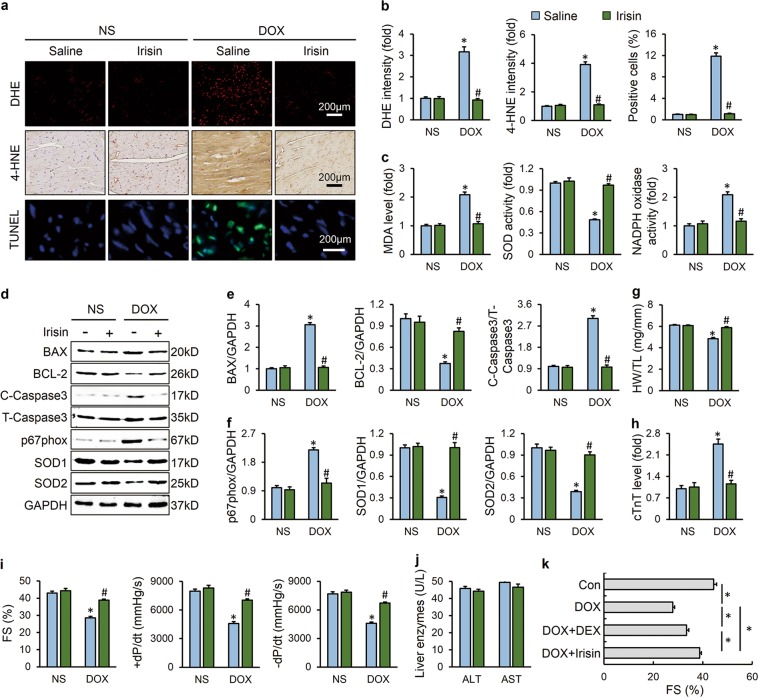
FNDC5/Irisin was a potential therapeutic agent against DOX-induced cardiotoxicity. **a**, **b** Representative DHE, 4-HNE, TUNEL staining images and statistical results (*n* = 6). **c** Quantitative results of myocardial MDA levels and SOD, NADPH oxidase activities (*n* = 6). **d**–**f** Western blots and statistical results (*n* = 6). **g** Statistical results of the heart weight/tibia length ((HW/TL) (*n* = 8). (**h**) Serum cTnT levels (*n* = 6). **i** Echocardiographic and hemodynamic parameters (*n* = 8). **j** Serum liver enzymes levels (*n* = 8). **k** Fractional shortening (FS) of mice as determined via echocardiography (*n* = 8). Values represent the mean ± SEM. *P < 0.05 versus the corresponding normal saline (NS) group mice treated with saline, ^#^*P* < 0.05 versus DOX-treated mice treated with irisin. In figure (**k**), *P < 0.05 versus the matched group

To enhance the clinical impact of our current work, we investigated whether infusion of exogenous irisin would attenuate DOX-induced cardiotoxicity. Administration of irisin markedly decreased DOX-triggered ROS overproduction and cardiomyocyte apoptosis (Fig. 8a–f). The decreased HW/TL and elevated serum cTnT level were both alleviated by irisin supplementation (Fig. 8g, h). Besides, the impaired heart function caused by DOX was also improved (Fig. 8i). More importantly, we found that systemic administration of irisin showed no hepatic toxicity in mice, as evaluated by the serum concentrations of liver enzymes (Fig. 8j). DEX is the only cardioprotective agent proven to be effective in the treatment of DOX-induced cardiotoxicity, we then compared the efficiency between irisin and DEX. As shown in Fig. 8k, irisin (12 nmol/kg/day) worked better than DEX (60 mg/kg/week) in mice.

In addition to acute cardiac effects, von Hoff et al. observed that DOX therapy was also associated with chronic cardiotoxicity that occurred in about 1.7% of the patients [49]. We next assessed whether FNDC5 overexpression could alleviate cardiac dysfunction in a chronic model of DOX-induced cardiotoxicity. As shown in Fig. S6a-b, FNDC5 overexpression ameliorated cardiac dysfunction and HW reduction in response to chronic DOX treatment. Moreover, we observed that FNDC5 overexpression also mitigated the mortality in response to repeated DOX injection (Fig. S6c). In line with the effect in acute model, we found that irisin was more effective than DEX in chronic cardiotoxicity (Fig. S6d). Considering the fact that long-term use of high-doses of DEX resulted in severe side effects, such as hepatic toxicity, occurrence of second malignancy and early death, we then investigated whether combined use of DEX with irisin could decrease the usage of DEX. As shown in Fig. S6e, combined use of DEX (30 mg/kg/week) with irisin (6 nmol/kg/day) could decrease the dosage of DEX, thereby might alleviate DEX-triggered side effects. All the data proved that FNDC5/Irisin was a potential therapeutic agent against DOX-induced cardiotoxicity.

## Discussion

In the present study, we found that DOX treatment decreased myocardial level of FNDC5 and cardiac-specific overexpression of FNDC5 or irisin supplementation alleviated oxidative stress and cardiomyocyte apoptosis in DOX-induced cardiotoxicity in mice. Consistent with the data in vivo, irisin treatment exerted similar beneficial effects on DOX-induced oxidative damage and cardiomyocyte apoptosis in H9C2 cells. Mechanistically, we identified that FNDC5/Irisin activated AKT/mTOR signaling and decreased DOX-induced cardiomyocyte apoptosis, and moreover, we provided direct evidence that the anti-oxidant effect of FNDC5/Irisin was mediated by the AKT/GSK3β/FYN/Nrf2 axis in an mTOR-independent manner. And we also demonstrated that HSP20 was responsible for the activation of AKT caused by FNDC5/Irisin. Based on these findings, we supposed that FNDC5/Irisin was a potential therapeutic agent against DOX-induced cardiotoxicity.

FNDC5/Irisin is a recently identified exercise-induced myokine, which improves systemic metabolism via increasing energy expenditure and serves as an appealing therapeutic target for treatment of metabolic disorders [18, 20, 30]. However, emerging evidence identified an essential role of FNDC5/Irisin in the regulation of heart development and function. Farzaneh et al. found that the mRNA level of Fndc5 was increased during the process of cardiac differentiation, and overexpression of FNDC5 significantly increased whereas Fndc5 deficiency decreased cardiomyocyte differentiation rate of mouse embryonic stem cells [50–52]. In addition, several researches have proved that FNDC5/Irisin played a pivotal role in protecting the heart against ischemia/reperfusion injury and could be a potentially candidate marker for myocardial infarction [23, 53, 54]. Irisin supplementation could also attenuate collagen deposition and ventricular function impairment in diabetic hearts [55]. Recently, results from Li et al. suggested that irisin alleviated pressure overload-induced cardiac hypertrophy in mice [24]. Previous studies indicated that DOX showed a specific affinity to the heart and the heart is a preferential target of DOX [56, 57]. In addition, DOX treatment destroyed myocardial redox homeostasis, and resulted in vacuolar degeneration and apoptosis of cardiomyocytes [4]. However, there is no available data about the role of FNDC5/Irisin in DOX-induced cardiotoxicity. In the current study, we observed that *Fndc5* deficiency at baseline elicited oxidative stress and apoptosis in H9C2 cells, imitating the phenotype of DOX-induced cardiomyopathy in vitro. Cardiac-specific overexpression of FNDC5 or irisin supplementation alleviated oxidative stress and cardiomyocyte apoptosis in DOX-induced cardiotoxicity in vivo and in vitro. Skeletal muscle has previously been recognized as the main source of FNDC5/Irisin, however, in this study, we observed that the expression of FNDC5 was more abundant in the myocardium, which was markedly reduced in response to DOX. Numerous studies identified the heart as an endocrine organ and named the secretomes from the heart as ‘cardiokines’, which are increasingly recognized as essential regulators of cardiac physiology and pathology [58]. Previous studies also suggested the existence of irisin-specific receptor on the membrane of cardiomyocytes [47]. Hence, we dare to speculate that FNDC5/Irisin might be one of the ‘cardiokines’ and exert the cardioprotective effect in autocrine or paracrine manners, however, this hypothesis must be experimentally validated.

Previous studies revealed that AKT inactivation was responsible for DOX-induced cardiotoxicity, and enhanced AKT phosphorylation promoted cardiomyocyte survival and prevented DOX-induced cardiac dysfunction [11, 12]. The results from our study showed that FNDC5 overexpression in murine hearts could activate AKT/mTOR and exert protective effects on cardiomyocyte apoptosis, which is consistent with a previous study suggested that mTOR inhibition was the major contributor to DOX-induced cardiotoxicity [14]. Oxidative stress is regarded as another main contributor to DOX-induced cardiotoxicity. DOX treatment inhibits mitochondrial biosynthesis-related gene expression, disrupts of mitochondrial structure and function, and hampers normal mitochondrial metabolism and repairment, which eventually contributes to increased ROS production. Besides, upregulation of nitric oxide synthase, enhanced NADPH oxidase activity and formation of DOX-iron complexes are also responsible for DOX-triggered ROS generation [3, 5, 8]. We observed that FNDC5/Irisin overtly decreased DOX-elicited oxidative stress damage, which could be reversed by AKT inhibition, but not rapamycin. Previous studies reported that AKT dephosphorylation resulted in GSK3β/FYN activation and FYN nuclear accumulation, which then promoted the nuclear exclusion of Nrf2 and its degradation in cytoplasm [15–17]. In this study, we found that FNDC5/Irisin alleviated oxidative damage via AKT/GSK3β/FYN/Nrf2 axis. Nrf2 is generally considered as the key transcription factor against oxidative stress by enhancing anti-oxidant gene expression, including HO-1. HO-1 or its metabolites-bilirubin was previously shown to inhibit the expression of NADPH oxidase subunits and NADPH oxidase activity in vivo and in vitro [59–61]. Furthermore, Li et al. previously demonstrated that HO-1 activation inhibited p67phox expression and prevented its membrane translocation [62]. Consistent with these data, we found that FNDC5 overexpression or irisin supplementation significantly inhibited NADPH oxidase activity and decreased p67phox subunit expression. Collectively, our results defined FNDC5/Irisin as an antioxidant to prevent cardiomyocytes from oxidative damage.

However, the pathway through which FNDC5/Irisin activated AKT remains unclear in this study. PI3K was previously reported to be involved in irisin-mediated activation of AKT, however, we found that irisin treatment unaffected the PI3K activity in response to DOX, but increased the expression of HSP20. Fan et al. suggested that HSP20 might block the action of phosphatases on AKT dephosphorylation, and HSP20 overexpression could effectively suppress oxidative stress as well as cardiomyocyte apoptosis [12]. HSPs, as molecular chaperones, have been extensively investigated on protection of DOX-induced cardiotoxicity. HSP27 has been regarded as the endogenous anti-oxidant and anti-apoptotic protein against DOX and attenuated DOX-induced cardiac dysfunction in mice [63]. Our present study found that HSP20 was responsible for the beneficial role of irisin, and Hsp20 deficiency abolished irisin-mediated activation of AKT. In addition, we compared the efficiency between irisin and DEX in the protection of DOX-induced cardiotoxicity, and it was worth noting that irisin worked better than DEX with no hepatic toxicity detected, and combined irisin with DEX treatment could decrease the dosage of DEX in mice. The data from this study provided evidence that it was possible using irisin as an adjuvant or alternative agent for the therapy of DOX-induced cardiotoxicity. This notion was further supported by the fact that irisin administration enhanced tumor sensitivity to DOX [64].

In conclusion, FNDC5/Irisin might be a potential therapeutic agent against DOX-induced cardiotoxicity.

## Supplementary information

Supplementary materials
