# Supplementary material for: FNDC5 alleviates oxidative stress and cardiomyocyte apoptosis in doxorubicin-induced cardiotoxicity via activating AKT
Source: Cell Death Differ. 2019 Jun 17;27(2):540–55. doi: 10.1038/s41418-019-0372-z (PMC7206111; doi:10.1038/s41418-019-0372-z)
Supplement: Supplementary file 1 — Supplementary materials [file 41418_2019_372_MOESM1_ESM.docx]

**FNDC5 alleviates** **oxidative stress and cardiomyocyte apoptosis in** **doxorubicin-induced cardiotoxicity via activating AKT**

Xin Zhang^*^, Can Hu^*^, Chun-Yan Kong, Peng Song, Hai-Ming Wu, Si-Chi Xu, Yu-Pei Yuan, Wei Deng,

Zhen-Guo Ma & Qi-Zhu Tang

Department of Cardiology, Renmin Hospital of Wuhan University, Wuhan 430060, PR China

Cardiovascular Research Institute of Wuhan University, Wuhan 430060, PR China

Hubei Key Laboratory of Cardiology, Wuhan 430060, PR China

^*^ These authors contributed equally to this work.

Corresponding author:

**Qi-Zhu Tang and Zhen-Guo Ma,**

Department of Cardiology,

Renmin Hospital of Wuhan University,

Cardiovascular Research Institute,

Hubei Key Laboratory of Cardiology,

Wuhan University at Jiefang Road 238, Wuhan 430060, PR China

Tel.: +86 27 88073385; Fax: +86 27 88042292.

E-mail: [qztang@whu.edu.cn](mailto:qztang@whu.edu.cn) (Qi-Zhu Tang) and [zhengma@whu.edu.cn](mailto:zhengma@whu.edu.cn) (Zhen-Guo Ma)

Running title: FNDC5 alleviates DOX-induced cardiotoxicity

The authors declare no conflict of interest.

**Supplementary Table: Primer Sequences used in our study**

| **Gene** | **Species** |  | **Sequence** |
| --- | --- | --- | --- |
| *Gapdh* | Mouse | Forward | ACTCCACTCACGGCAAATTC |
|  |  | Reverse | TCTCCATGGTGGTGAAGACA |
| *Fndc5* | Mouse | Forward | ATGAAGGAGATGGGGAGGAA |
|  |  | Reverse | GCGGCAGAAGAGAGCTATAACA |
| *Nqo1* | Rat | Forward | TATCCTTCCGAGTCATCTCTAGCA |
|  |  | Reverse | TCTGCAGCTTCCAGCTTCTTG |
| *Gclc* | Rat | Forward | AAACACGCCTTCCTTCCCATTG |
|  |  | Reverse | AGTAA AGTGGCACAGGAGCGAG |
| *Gclm* | Rat | Forward | AATCTTGCCTTCCTTCCCATTG |
|  |  | Reverse | GGCTTCAATGTCAGGGATGCTTTC |
| *Nrf2* | Rat | Forward | CGAGATATACGCAGGAGAGGTAAGA |
|  |  | Reverse | GCTCGACAATGTTCTCCAGCTT |
| *Hsp20* | Rat | Forward | ATTTTTCTGTGCTGG |
|  |  | Reverse | CAGGAGACAGTGCAGAGG |
| *Hsp27* | Rat | Forward | CCTCTTCGATCAAGCTTT'CG |
|  |  | Reverse | CTGGAGGGAGCGTGTATTTC |
| *Hsp60* | Rat | Forward | AGGCATGAAGTTTGATAGAGG |
|  |  | Reverse | TTFFCAATTTCAAGAGCAGG |
| *Hsp70* | Rat | Forward | CCGCGTGATGGACGTGTAG |
|  |  | Reverse | AGATGGTCACTGTCTGCCATGTGGG |
| *Hsp72* | Rat | Forward | GCAGTCGGACATGAAGCACT |
|  |  | Reverse | CGAACAGAGAGTCGATCTGC |
| *Hsp90* | Rat | Forward | GTCCCGGTGCGGTTAGTCACG |
|  |  | Reverse | TTGGGTCTGGGTTTCTCAGGC |
| *Fndc5* | Rat | Forward | CAGCAGAAGAAGGATGTGAG |
|  |  | Reverse | GGCAGAAGAGAGCTATGACA |
| *Gapdh* | Rat | Forward | GACATGCCGCCTGGAGAAAC |
|  |  | Reverse | AGCCCAGGATGCCCTTTAGT |


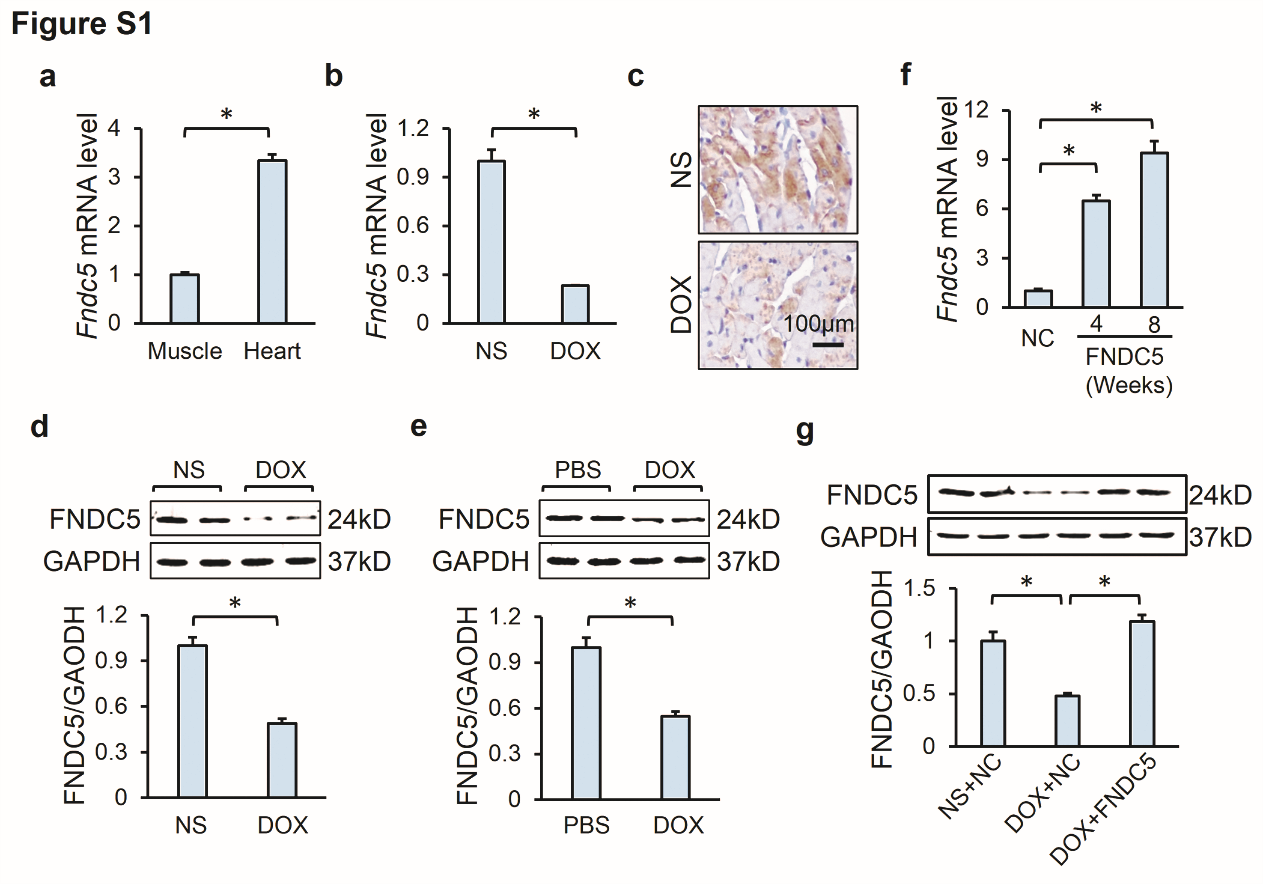


**Figure S1. FNDC5 expression was decreased in doxorubicin (DOX)-treated murine hearts and cardiomyocytes.** (a) Relative *Fndc5* mRNA level in murine hearts and skeletal muscle (n=12). (b) Alteration of myocardial *Fndc5* mRNA level in response to DOX (n=9). (c) Representative immunohistochemistry images of FNDC5 in murine hearts (n=6). (d-e) Western blot and quantitative results of FNDC5 in DOX-treated hearts and H9C2 cardiomyocytes (n=6). (f) The mRNA level of FNDC5 in hearts after AAV9 injection at the indicated times (n=5). (g) Protein expression of FNDC5 in murine hearts in response to DOX insult (n=6). Values represent the mean±SEM. **P*<0.05 versus the corresponding group.


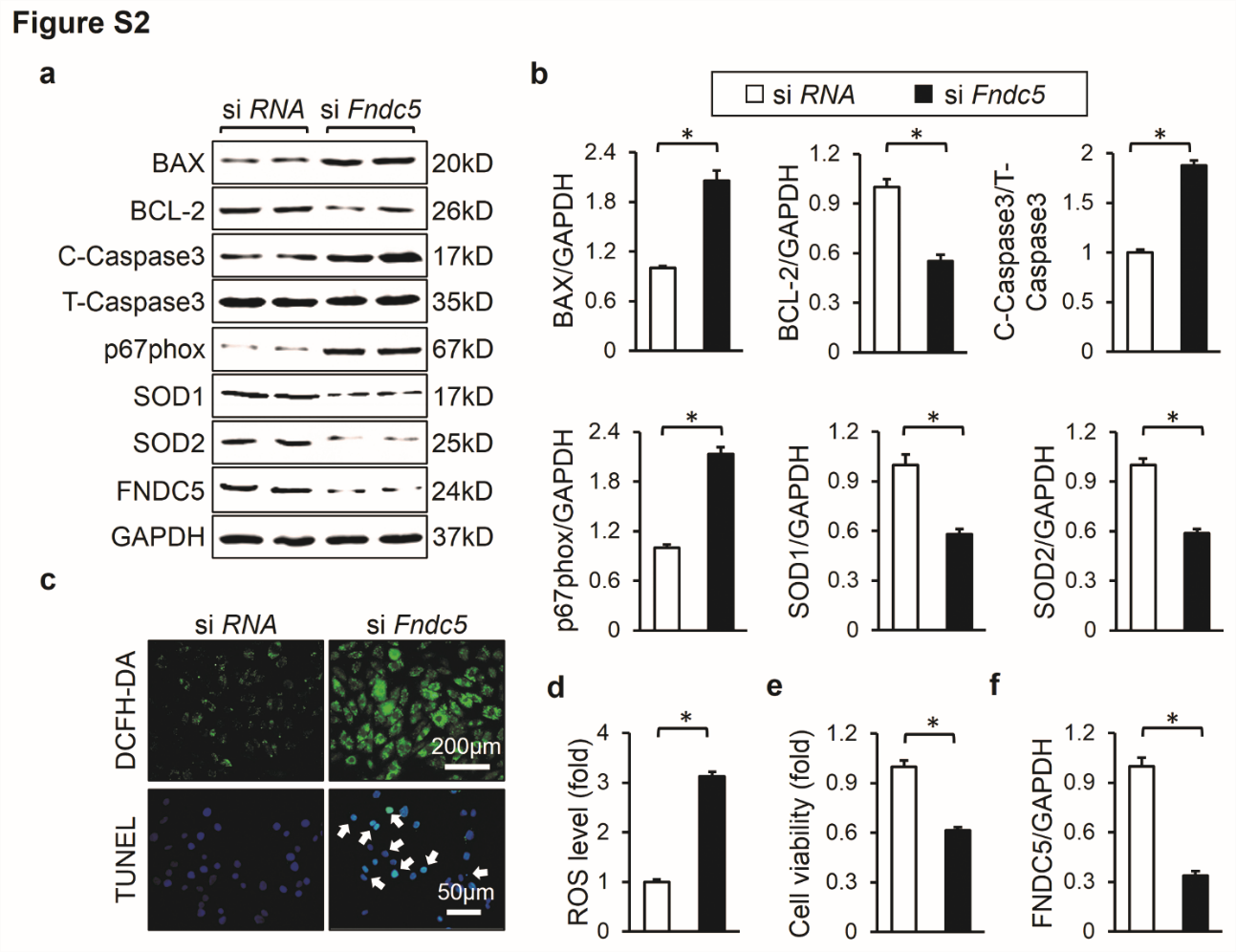


**Figure S2. *Fndc5* deficiency resulted in increased oxidative damage and apoptosis in H9C2 cells under basal conditions.** (a-b) Representative western blots and quantitative results (n=6). (c) Representative images of DCFH-DA detection and TUNEL staining (n=6). (d) Quantitative results of ROS level (n=6). (e) Cell viability results by CCK-8 assay (n=5). (f) Quantitative results of si *Fndc5* efficiency (n=6). Values represent the mean±SEM. **P*<0.05 versus the corresponding group.


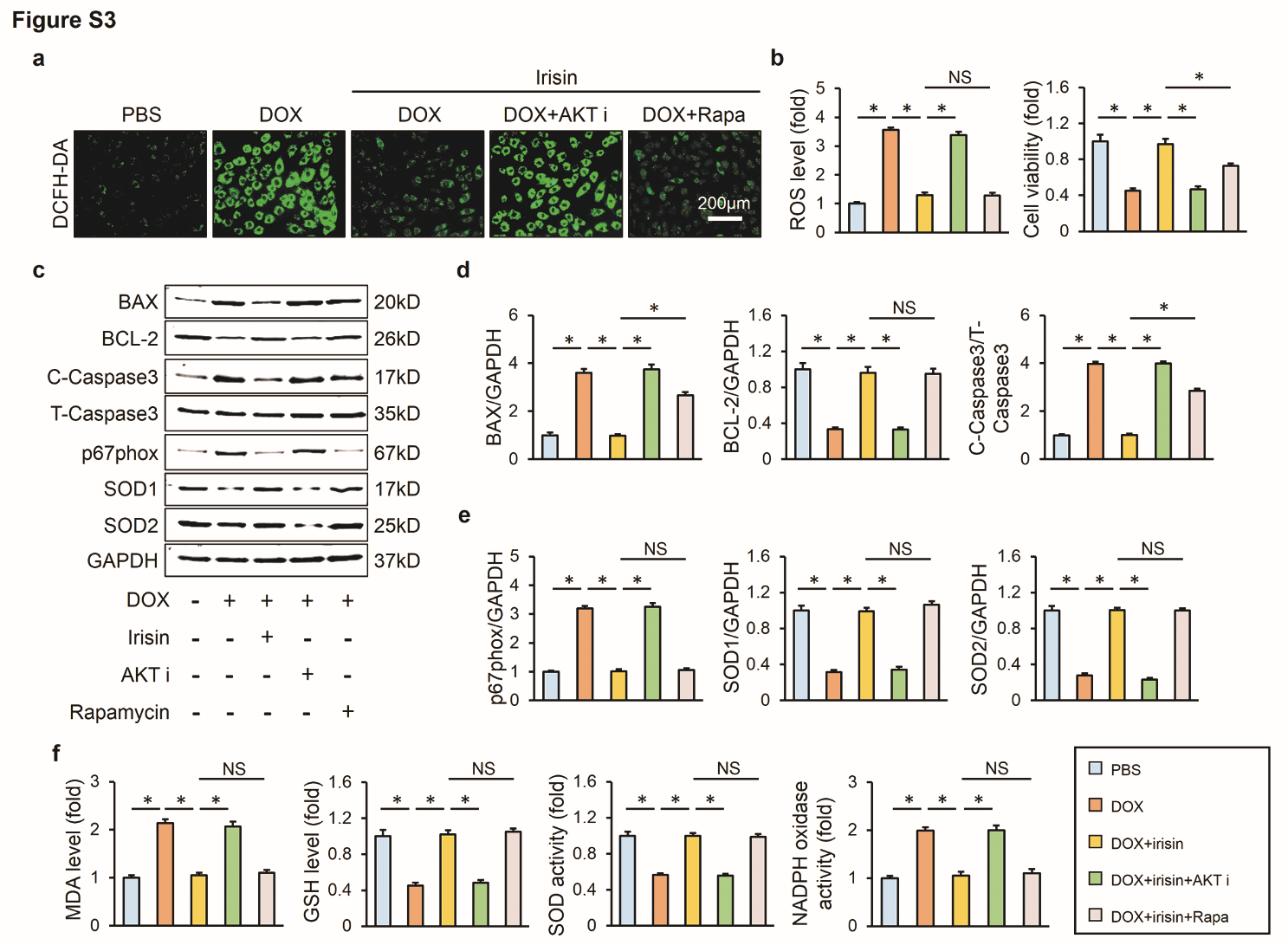


**Figure S3. Rapamycin partly reversed irisin-mediated anti-apoptotic effects, with no influence on the beneficial effects on oxidative damage.** (a-b) Representative DCFH-DA images, CCK-8 assay and quantitative results (n=6). (c-e) Representative western blots and quantitative results (n=6). (f) Quantitative results of MDA, GSH levels and SOD, NADPH oxidase activities in cultured H9C2 cells (n=6). Values represent the mean±SEM. **P*<0.05 versus the corresponding group, NS means no significance.


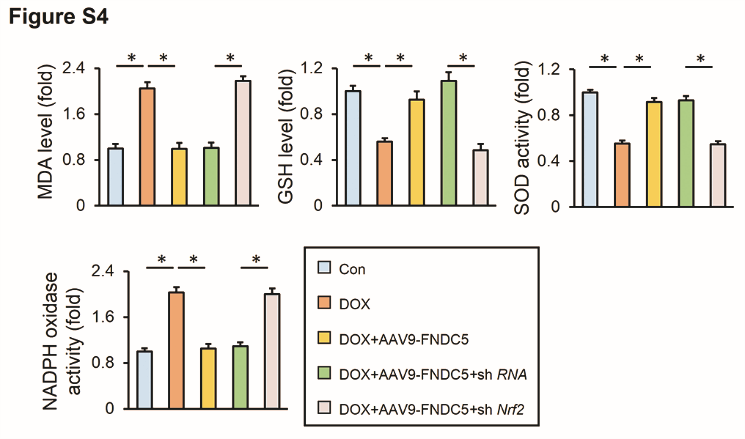


**Figure S4. FNDC5 lost its anti-oxidant effect in *Nrf2*-deficient mice.** Quantitative results of myocardial MDA, GSH levels and SOD, NADPH oxidase activities (n=6). Values represent the mean±SEM. **P*<0.05 versus the corresponding group.


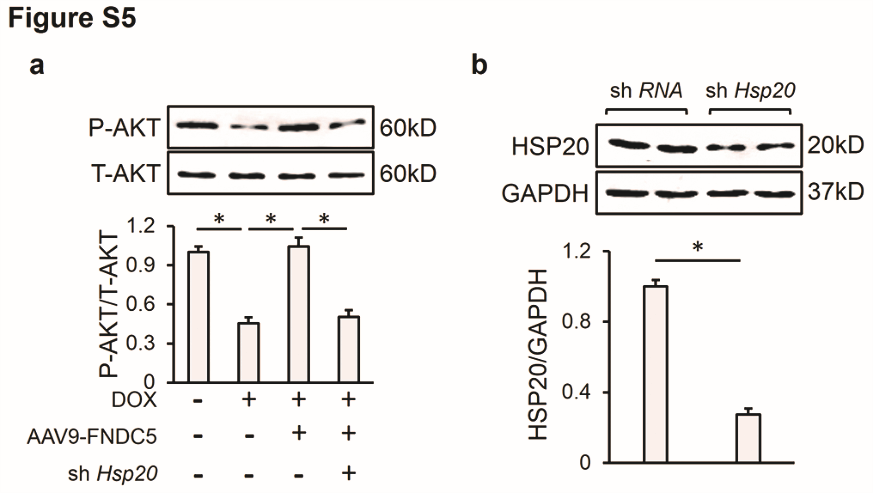


**Figure S5. HSP20 was responsible for FNDC5-mediated activation of AKT in mice.** (a) Western blots and quantitative data (n=6). (b) Efficiency of sh *Hsp20* detected by western blots (n=6). Values represent the mean±SEM. **P*<0.05 versus the matched group.


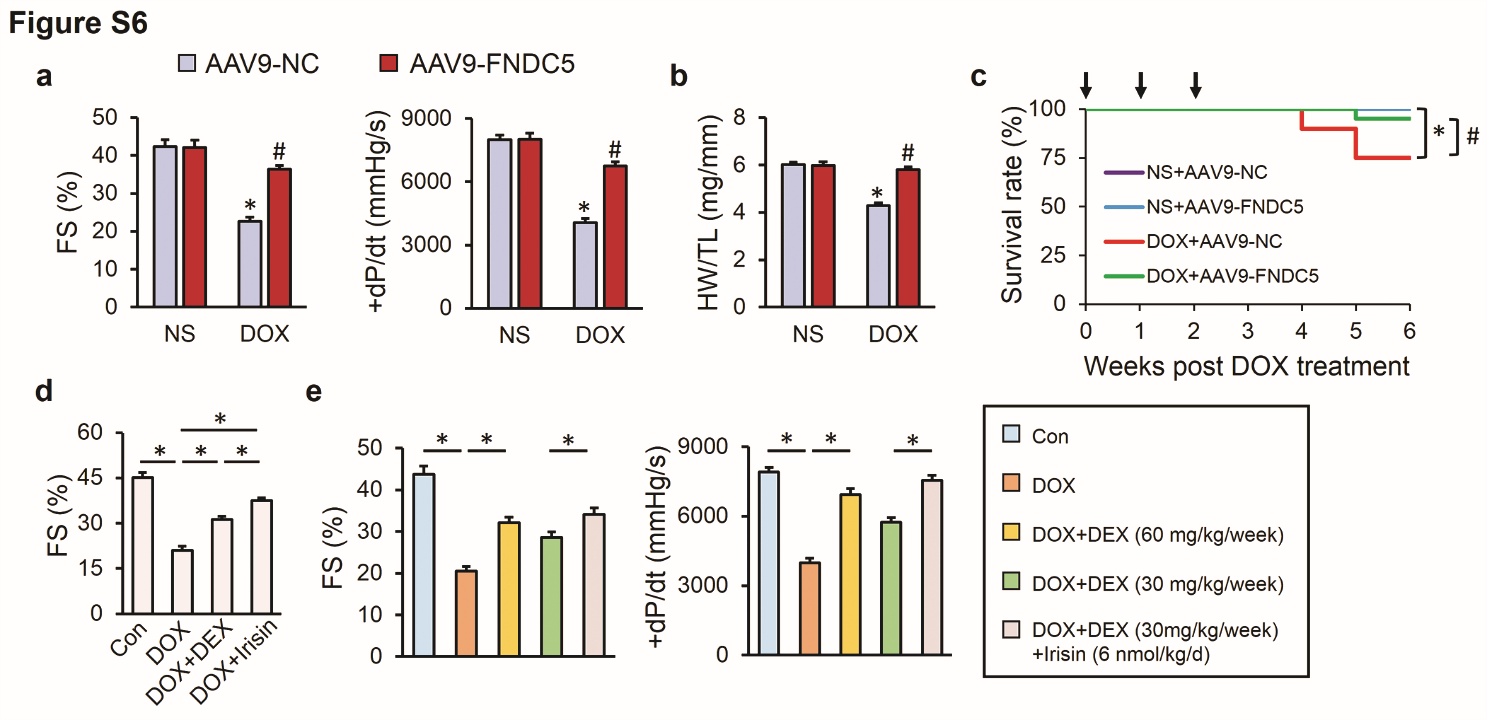


Figure S6. The protective role of FNDC5/Irisin in chronic model of DOX-induced cardiotoxicity. (a) Echocardiographic and hemodynamic parameters after FNDC5 overexpression (n=8). (b) Statistical results of the heart weight/tibia length (HW/TL) after FNDC5 overexpression (n=8). (c) Survival rate in mice that exposed to repeated injections of low dosage DOX (5 mg/kg, i.p., once a week, the total cumulative dose is 15 mg/kg) (n=20). (d) Fractional shortening (FS) of mice as determined via echocardiography (n=8). (e) Echocardiographic and hemodynamic parameters (n=8). Values represent the mean±SEM. **P*<0.05 versus the corresponding normal saline (NS) group mice injected with negative control (NC) adeno-associated virus 9, #*P*<0.05 versus DOX-treated mice injected with AAV9-NC. In figure d-e, **P*<0.05 versus the matched group.
